# Natural vertical transmission of dengue virus in *Aedes aegypti* and *Aedes albopictus*: a systematic review

**DOI:** 10.1186/s13071-018-2643-9

**Published:** 2018-02-01

**Authors:** Victor Henrique Ferreira-de-Lima, Tamara Nunes Lima-Camara

**Affiliations:** 1 Postgraduate Program at Institute of Tropical Medicine, University of São Paulo, Av. Dr. Enéas de Carvalho Aguiar, 470, Jardim America, São Paulo, SP 05403-000 Brazil; 2 Department of Epidemiology, School of Public Health, University of São Paulo, Av. Dr. Arnaldo, 715, Cerqueira César, São Paulo, SP 03178-200 Brazil

**Keywords:** Vertical transmission, Dengue, Chikungunya, Zika, *Aedes*, Vectors

## Abstract

Dengue is of great concern in various parts of the world, especially in tropical and subtropical countries where the mosquito vectors *Aedes aegypti* and *Aedes albopictus* are present. The transmission of this virus to humans, by what is known as horizontal transmission, occurs through the bite of infected females of one or other of the two mosquito species. Furthermore, an infected female or male parent, by what is known as vertical transmission, can transfer this arbovirus to some part of their offspring. Considering that vertical transmission may represent an important strategy for maintaining the circulation of arboviruses in nature, the verification of this phenomenon worldwide is extremely important and necessary to better understand its dynamic. In the present study, we conducted a literature review of the presence of natural vertical transmission of dengue virus in *Ae. aegypti* and *Ae. albopictus* worldwide. Searches were conducted in MEDLINE, sciELO and Lilacs and all the studies published in Portuguese, English and Spanish were read, evaluated and organized by mosquito species, serotype and the location at which the samples were collected. Forty-two studies were included in accordance with the exclusion criteria and methodology. The presence of natural vertical transmission in *Ae. aegypti* and *Ae. albopictus* was most clearly evidenced by dengue virus in endemic countries, especially in those in South America and Asia. Despite several African countries being considered endemic for dengue, there is a lack of publications on this subject on that continent, which highlights the importance of conducting studies there. Furthermore, the finding of natural vertical transmission in *Ae. albopictus* in countries where this species is not yet incriminated as a vector is of great concern as it demonstrates the circulation of this virus in populations of *Ae. albopictus* and alerts to the possibility of some other mosquito species playing a role in the transmission dynamics of this arbovirus. Parallel to this, the small number of studies of natural vertical transmission of chikungunya and Zika virus in the world may be explained by the recent entry of these arboviruses into most of the countries concerned.

## Background

Dengue is a viral infection caused by four antigenically distinct serotypes (DENV-1, DENV-2, DENV-3 and DENV-4) and which belongs to the genus *Flavivirus* (family Flaviviridae) [1, 2]. It is widespread in more than one hundred tropical and subtropical countries in Asia, Africa and the Americas [3], where conditions of temperature and humidity favor the proliferation of mosquito vectors [2, 4].

Dengue is considered the most serious re-emerging viral disease transmitted by arthropods with an estimated almost 390 million new dengue infections every year worldwide [5]. The first reports of a disease with clinical symptoms similar to those of dengue is in a Chinese Medical Encyclopedia from the Jin Dynasty (AD 265–420) whereas the first main epidemics described with symptoms compatible with those of dengue fever date from the eighteenth century on three continents: Asia, Africa and North America. Only since the1940s was the dengue virus isolated from patients, with a subsequent conclusion drawn of the presence of four serotypes [2].

Today, more than 2.5 billion people live in areas of risk of infection and approximately 96 million cases present clinical manifestations of dengue, such as fever, rash, eye pain, arthralgias, myalgias and hemorrhage [2, 5]. Dengue has a significant economic impact on many tropical and subtropical countries [2] and the factors responsible for its dramatic re-emergence as a global health problem are complex. These factors are probably related to the increasing global human population as well as to the unplanned and uncontrolled urbanization which established ideal conditions for the transmission of the dengue virus (DENV) [2].

The DENV is mainly transmitted to humans through the bite of infected female mosquitoes of the *Aedes* genus, this process being called horizontal transmission [6]. By this mechanism, the mosquito becomes infected when it ingests blood from a viremic host. The extrinsic incubation period (EIP) is defined as the time between the ingestion of the infected blood by a susceptible mosquito and the presence of infective viral particles in its salivary secretions. After this period, the insect is capable of transmitting the virus to a new vertebrate host [7]. However, a phenomenon known as vertical transmission relates to the transfer of some arboviruses from the infected female or male parent to some part of their offspring within the ovary or during oviposition [8–11].

Considering that natural vertical transmission may represent an important strategy to maintain the circulation of several arboviruses in the mosquito vector population, the verification of this phenomenon worldwide is extremely important and necessary to better understand the dynamics of the transmission of these pathogens. As part of the present study, we conducted a literature review on the presence of natural vertical transmission of DENV in *Ae. aegypti* and *Ae. albopictus* worldwide.

## Data collection

Searches were conducted in the indexers of scientific articles MEDLINE (http://www.ncbi.nlm.nih.gov/pubmed), sciELO (http://www.scielo.org) and Lilacs (http://lilacs.bvsalud.org/) using the combination of the terms: “*Aedes aegypti*”, “*Aedes albopictus*”, “dengue”, and “vertical transmission”. All the studies published in Portuguese, English and Spanish were read, evaluated and organized by mosquito species, serotype and location where the samples were collected. To avoid the inadvertent exclusion of any studies by using these pre-determined keywords, manual searches of the articles cited in the reference lists were also conducted in order to include the largest possible number of publications. Data published before 31st August 2017 were extracted.

All articles were grouped according to the serotype (DENV-1, DENV-2, DENV-3, DENV-4) and the location at which the samples were collected (Country and State/ Island/ Province). Only studies that demonstrated natural vertical transmission in *Ae. aegypti* and *Ae. albopictus*, that is, the transference of the virus from a male or female parent to its offspring [8], were used whereas data originating only under laboratory conditions were excluded from the analysis.

**Fig. 1:**
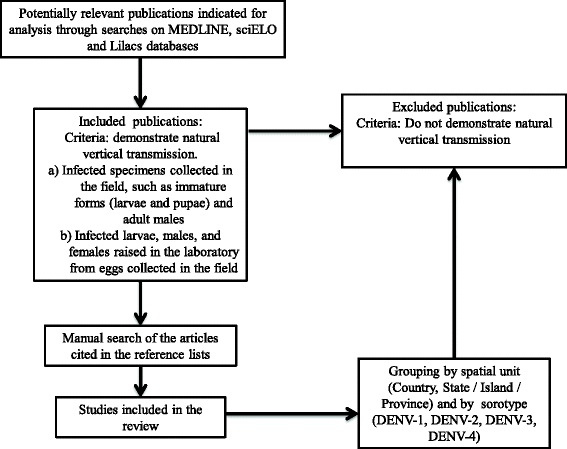
Data collection for the literature review. Flowchart for the preparation of the literature review of natural vertical transmission of DENV in *Ae. aegypti* and *Ae. albopictu*s worldwide

In this study, we considered natural vertical transmission as that observed in infected specimens collected in the field, such as immature forms (larvae and pupae) and adult males. Infected larvae, males, and females raised in the laboratory from eggs collected in the field were also considered as vertical transmission. Vertical transmission demonstrated through the infection of females by intrathoracic injection or oral feeding with infected blood under artificial laboratory conditions was considered experimental and has not been used in this study (Fig. 1).

## Vertical transmission worldwide

Searches conducted in the MEDLINE, SciELO and Lilacs databases using the combination of the pre-defined terms provided 96 articles, but only 32 studies were considered relevant in the light of the exclusion criteria. After an extensive manual examination of the reference lists of all the articles considered relevant, 10 more publications were included, totaling 42 studies.

Vertical transmission of DENV has been demonstrated in seven and three consecutive generations of *Ae. aegypti* and *Ae. albopictus*, respectively, under laboratory conditions [12, 13], which may contribute to its perpetuation in the populations of these vectors under adverse conditions for horizontal transmission [8, 13]. The number of studies reporting the presence of the natural vertical transmission of DENV in *Ae. aegypti* and *Ae. albopictus* confirms the reality of this phenomenon.

**Fig. 2:**
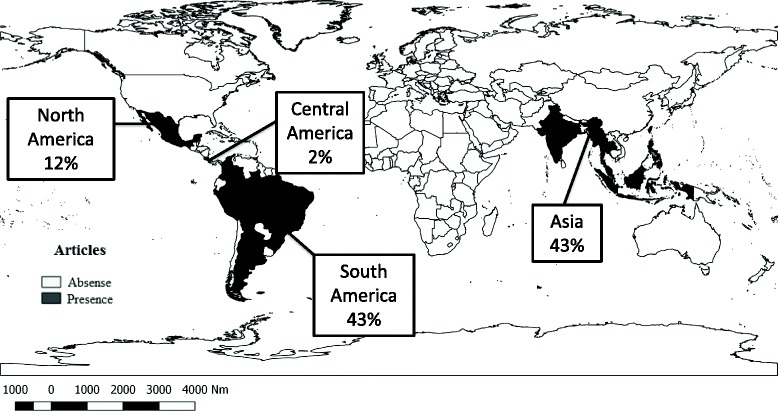
Vertical transmission worldwide. Distribution of publications on the natural vertical transmission of DENV in *Ae. aegypti* and *Ae. albopictus*, by region

South America and Asia were the continents with the highest number of published articles (*n* = 36; 86%), followed by North America (*n* = 5; 12%) and Central America (*n* = 1; 2%) (Fig. 2). In Asia, most of the studies of natural vertical transmission of DENV in *Ae. aegypti* and *Ae. albopictus* were published in India (6/18; 33%) and Thailand (4/18; 22%). In North America, Mexico was the only country where this phenomenon was demonstrated for these species whereas in Central America it was only evidenced in Costa Rica. All the countries in which the natural vertical transmission of DENV has been confirmed are considered endemic for this arbovirus.

In South America, most of the published studies were undertaken in Brazil (13/18; 72%), indicating the importance of this country in the study of the dynamics of these arboviruses in mosquito vector populations. However, these studies are not well distributed geographically, as they were mostly (6/13; 46%) conducted in Minas Gerais state, Southeastern Brazil, indicating the importance of carrying out such studies in other states where *Ae. aegypti* and *Ae. albopictus* are present.

South America and Asia are the largest producers of knowledge on this subject and this might be related to the high number of endemic countries on the two continents. The low number of studies undertaken in both North and Central America indicates the need for further studies to better understand the dynamics of this phenomenon in these areas.

It is important to point out that the discrepancy observed in the number of studies on vertical transmission in all the continents does not necessarily mean that areas with a larger amount of published scientific articles are the most epidemiologically relevant sites for this virus. Endemic areas for dengue, with intense transmission of DENV, such as African countries, present a lack of studies on natural vertical transmission in *Ae. aegypti* and *Ae. albopictus*.

## The natural vertical transmission of DENV in Asia

In the 80s Khin & Than [14] investigated the possibility of DENV being naturally vertically transmitted in *Ae. aegypti*. In their study the authors suggested the natural vertical transmission of the DENV-2 serotype in adult males of *Ae. aegypti* from Myanmar. However, its importance for the maintenance of the circulation of DENV serotypes under natural conditions was questioned by Watts et al. [15]. Even though the authors conducted analysis of more than 5 thousand larvae, 30 pupae and 80 males of *Ae. aegypti* in Bangkok, Thailand, none of the pools were considered positive for any DENV serotype, whereas infected *Ae. aegypti* females with DENV-2 were captured in the same area. Hutamai et al. [16] sought to evidence the natural vertical transmission of the dengue virus in *Ae. aegypti* and *Ae. albopictus*. These authors collected samples from 17 dengue re-epidemic areas in Chiang Mai and Lampang Provinces, North of Thailand, raised immature stages under laboratory conditions up to the adult stage, and tested for dengue viral RNA, by a nucleic acid sequence-based amplification assay (NASBA). However, no infected specimen was found in the samples tested (9825 *Ae. aegypti* and 150 *Ae. albopictus*) thus corroborating the finding of Watts et al. [15].

Nevertheless, in 2011 this phenomenon was finally observed in Bangkok [17], when all four serotypes of the dengue virus were detected in adult males and females of *Ae. aegypti* reared continuously until adulthood in the laboratory. DENV-4 was the most prevalent serotype, followed by DENV-3, DENV-1 and DENV-2. The authors suggested that the demonstration of the presence of the dengue virus in immature forms in natural environments is essential for the rapid identification of the focal areas of this disease [17].

The possibility of the natural vertical transmission of DENV was first discussed in India in 1991 [18]; however, none of the pools of males of *Ae. aegypti* were positive. Nevertheless, since 2007, several studies have demonstrated the presence of natural vertical transmission of dengue serotypes, such as DENV-2 and DENV-3, in males and females of both *Ae. aegypti* and *Ae. albopictus* in this country [19–22].

Over the years, some other Asian countries such as Malaysia [23, 24], Singapore [25], Indonesia [26] and the Philippines [27] have demonstrated the presence of this phenomenon in their territories, but natural vertical transmission of DENV has not been observed in Taiwan [28].

## The natural vertical transmission of DENV in the Americas

The first study of the natural vertical transmission of DENV in the Americas provided information on this phenomenon in specimens from Trinidad and Tobago [29]. These authors suggested that despite the vertical transmission of DENV not occurring with great frequency, it could represent an important mechanism for the establishment of endemicity followed by extensive viral activity and consequent increase in the levels of immunity in human populations. This could be a possible relevant mechanism for the maintenance of the virus during periods of low precipitation as well. In addition, the natural vertical transmission of DENV has been demonstrated in *Ae. aegypti* from Bolivia [30], Argentina [31] and Peru [32], though the presence of the same phenomenon in the same species in Colombia was not clear [33].

Natural vertical transmission in Brazil was first described in *Ae. albopictus* from the state of Minas Gerais in 1993; DENV-1 was detected in its larval stages [34]. Over the years, other studies have demonstrated the natural vertical transmission of the other serotypes, except for DENV-4, in the same state. For example, Cecílio et al. [35] detected the vertical transmission of DENV-1 and DENV-2 in *Ae. aegypti* larvae and Vilela et al. [36] identified DENV-3 in naturally infected field-caught *Ae. aegypti* males and larvae in Pompeu and Belo Horizonte, respectively. Pessanha et al. [37] collected eggs of *Ae. aegypti* and *Ae. albopictus* in Belo Horizonte and after subsequent egg hatching, the larvae were tested for the presence of dengue virus and the authors detected the presence of DENV-1, DENV-2 and DENV-3, thus corroborating the findings of Vilela et al. [36]. More recently, Cecílio et al. [38] collected eggs of *Ae. aegypti* and *Ae. albopictus* and detected DENV-2 in four pools of *Ae. aegypti* larvae*.*

Other studies have corroborated the hypothesis of natural vertical transmission in Brazil. In Santos, São Paulo state, the DENV-3 serotype was detected in *Ae. albopictus* larvae [39]; DENV-2 and DENV-3 were found in *Ae. aegypti* and *Ae. albopictus* adults from Fortaleza, Ceará [40]. DENV-1 was isolated from a male of *Ae. aegypti* in Rio de Janeiro city [41]. In Cuiabá, Mato Grosso, Cruz et al. [42] detected the presence of DENV-4 in males and females of *Ae. aegypti* reared under laboratory conditions from eggs collected in the field. Natural vertical transmission of DENV has not been observed in Roraima [43]. In the state of Amazonas, northern Brazil, the analysis of 1816 specimens of *Ae. aegypti* (674 adults and 1142 immature forms) from 35 municipalities showed negative results but the authors attribute this outcome to the low number of specimens analyzed [44]. On the other hand, a more recent study performed in municipalities of the Amazonas state analyzed an almost 4000 *Ae. aegypti* larvae sample and reported 46% of infected pools. All the serotypes were detected, DENV 1 and 4 being present in all the municipalities investigated [45].

Additionally, the occurrence of natural vertical transmission of all the serotypes of the DENV virus has been described in Mexico. Ílbanez-Bernal et al. [46] conducted collections of males and females of *Ae. aegypti* and *Ae. albopictus* during a dengue outbreak in 1995 in Reynosa, Tamaulipas, and one pool of ten *Ae. albopictus* males was positive for DENV-2 and DENV-3. This was the first report of *Ae. albopictus* naturally infected with a dengue virus in North America [46]. Güther et al. [47] collected larvae of *Ae. aegypti* from Oaxaca and reared them under laboratory conditions up to the adult stage. The authors detected DENV-2, DENV-3 and DENV-4 in 2 pools of females from Tuxtepec and in 2 from Juchitán. In Acapulco, Guerrero, Martínez et al. [48] collected *Ae. aegypti* in an area where dengue had been reported (probable or confirmed cases) and of a total of 4146 *Ae. aegypti* adults tested, two pools of males were considered positive for DENV-1. Similarly, DENV-3 and DENV-4 were detected in *Ae. aegypti* males in the same city [49]. In Nueva Leon, a pool of four females of *Ae. albopictus* reared in the laboratory from the egg stage was considered positive for DENV. The authors could not, however, determine the serotype [50].

**Fig. 3:**
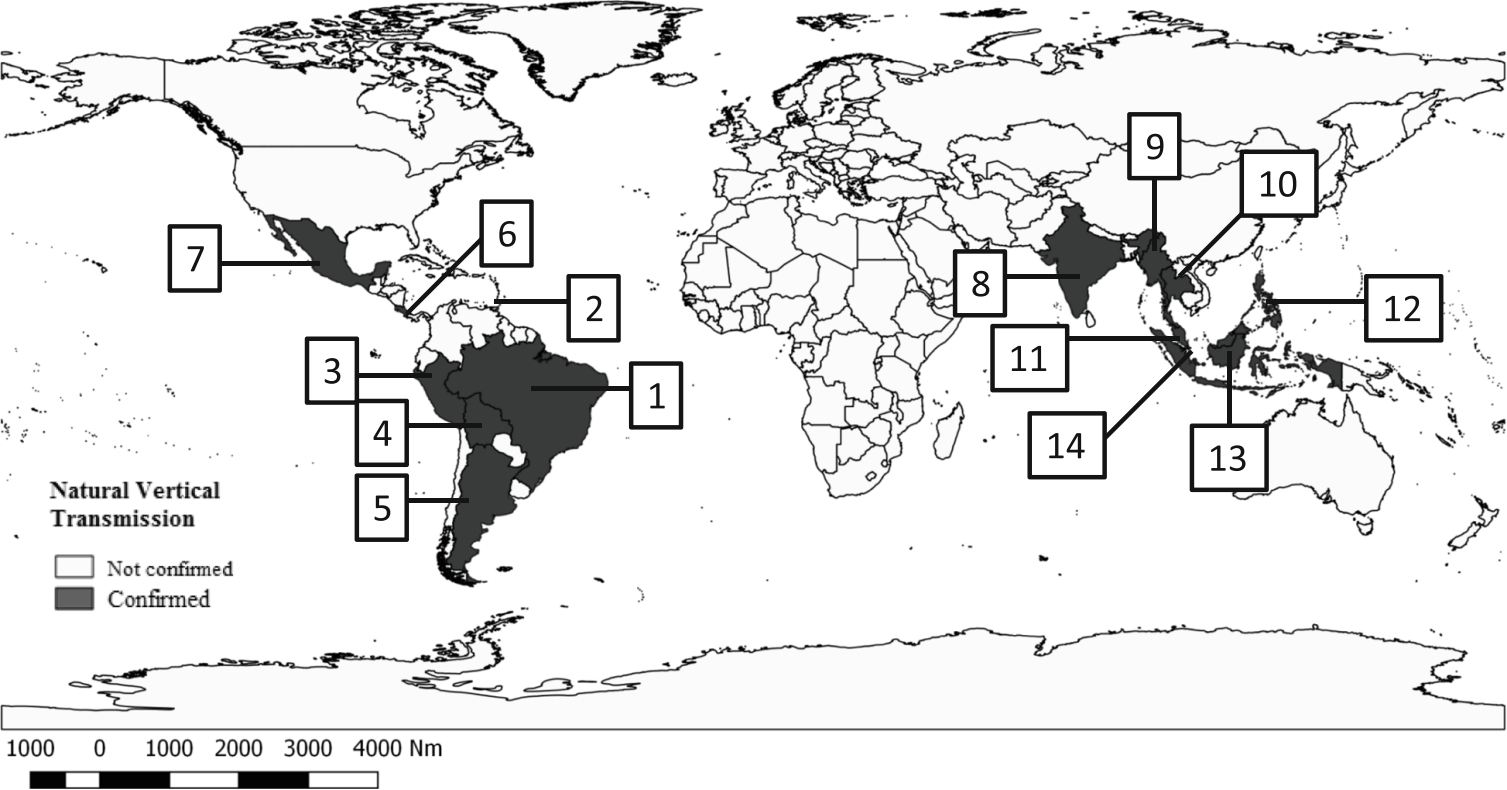
Confirmed cases of vertical transmission of DENV worldwide. Countries with confirmation of the natural vertical transmission of DENV in *Ae. aegypti* and *Ae. albopictus*. * Key*: 1, Brazil; 2, Trinidad and Tobago; 3, Peru; 4, Bolivia; 5, Argentina; 6, Costa Rica; 7, Mexico; 8, India; 9, Myanmar; 10, Thailand; 11, Malaysia; 12, Philippines; 13, Indonesia; 14, Singapore

The only research conducted in Costa Rica into vertical transmission demonstrated the presence of DENV in a pool of males of *Ae. albopictus* collected on the edge of an organic pineapple plantation, evidencing the natural circulation of this virus in this species [51]. A sketch-map of countries providing confirmation of this event in the Americas and Asia is presented in Fig. 3.

## Natural vertical transmission of other arboviruses: ZIKV and CHIKV

Zika is caused by a virus of the genus *Flavivirus* (family *Flaviviridae*) [52]. It was first isolated in a Rhesus monkey from the Zika Forest, Uganda, in 1947 during a yellow fever survey, and serological evidence of Zika virus (ZIKV) circulation in humans were reported in countries of East and West Africa as well [53]. In 2007, the first out-of-area epidemic occurred on the island of Yap, Micronesia, in the Pacific Ocean, and subsequently in October 2013, the virus reached French Polynesia, Oceania, where it caused a major outbreak of the disease [53]. The first country in the Americas to register autochthonous human cases of Zika and to confirm the circulation of the virus was Brazil, in early 2015, in the states of Bahia and Rio Grande do Norte [54–56]. Autochthonous cases have been reported in other countries such as Colombia, Bolivia, Venezuela, Paraguay, Peru, French Guiana, Haiti, El Salvador, Guatemala, Nicaragua, Cuba and Mexico [57]. Despite its low lethality, ZIKV has frequently been associated with the Guillain-Barré syndrome, microcephaly and other adverse consequences in neonates born of infected mothers [58–61].

Chikungunya virus (CHIKV) (genus *Alphavirus*; family *Togaviridae*) was first described in East Africa during an outbreak in Tanzania in 1952 and 1953. Fifty years later, in 2004, CHIKV emerged on the French island of Reunion, causing a great number of human cases [52, 62, 63]. Later, in 2006, chikungunya epidemics were recorded in India and some southeastern Asiatic countries, and in 2007, autochthonous cases of this arbovirus were reported in Italy [62]. At the end of 2013, CHIKV was isolated for the first time in the Americas, in the Caribbean region, and by 2014, South American countries such as French Guiana, Venezuela, Suriname and Brazil had already recorded autochthonous cases of chikungunya [64]. To date, the circulation of CHIKV is reported in Africa, Asia, Europe, South and Central America and in some southern states of the USA [64, 65].

Using the same methodology and exclusion criteria described above, we obtained two articles on natural vertical transmission of CHIKV and two on natural vertical transmission of ZIKV, totaling four articles. CHIKV was detected in *Ae. aegypti* males from Mexico [49] and in the same species from India [66]. Furthermore, evidence for the natural vertical transmission of ZIKV was reported in Brazil, initially in males of *Ae. aegypti* from Rio de Janeiro [67] and later in males and females of *Ae. albopictus*, from Camaçari, Bahia [68].

No relationship among endemism and vertical transmission was found for ZIKV or CHIKV, probably due to their recent introduction into the Americas and the low number of publications available for analysis.

## Conclusions

The confirmed presence of the natural vertical transmission of DENV in endemic countries may be related to a possible correlation between endemism and vertical transmission, suggesting that this phenomenon may represent an important mechanism for the establishment of endemicity [29]. This observation can also be explained by the greater interest of the scientific community towards a better understanding of dynamic transmission in countries that suffer from several arboviruses. In spite of some African countries being considered endemic for dengue, there is a lack of publications on this subject on that continent, which does not necessarily mean that natural vertical transmission does not occur in those countries and highlights the importance of conducting studies in the area. Parallel to this, the low number of studies on natural vertical transmission of CHIKV and ZIKV in the world may be explained by the recent entry of these arboviruses into the countries affected, causing severe epidemics and complications in the majority of them, especially in areas with previous circulation of DENV [69]. Nevertheless, the detection of this phenomenon in the Mexican, Indian and Brazilian populations of *Ae. aegypti* and *Ae. albopictus* highlights the importance of studying this occurrence in nature elsewhere. Additionally, despite being a vector of DENV, CHIKV and ZIKV in several countries of the world, *Ae. albopictus* is still regarded as a potential vector of these arboviruses in most countries of the Americas. The confirmation of the natural vertical transmission in this mosquito species is, therefore, of great importance as it demonstrates the circulation of these viruses in these populations and alerts to the possibility of a new vector acting in the transmission dynamics of these arboviruses.

## Abbreviations

CHIKV: chikungunya virus
DENV: dengue virus
EIP: extrinsic incubation period
ZIKV: Zika virus
